# Enhanced Auditory Neuron Survival Following Cell-Based BDNF Treatment in the Deaf Guinea Pig

**DOI:** 10.1371/journal.pone.0018733

**Published:** 2011-04-15

**Authors:** Lisa N. Pettingill, Andrew K. Wise, Marilyn S. Geaney, Robert K. Shepherd

**Affiliations:** 1 The Bionic Ear Institute, East Melbourne, Victoria, Australia; 2 Department of Otolaryngology, The University of Melbourne, Parkville, Victoria, Australia; 3 Living Cell Technologies Limited, Auckland, New Zealand; Claremont Colleges, United States of America

## Abstract

Exogenous neurotrophin delivery to the deaf cochlea can prevent deafness-induced auditory neuron degeneration, however, we have previously reported that these survival effects are rapidly lost if the treatment stops. In addition, there are concerns that current experimental techniques are not safe enough to be used clinically. Therefore, for such treatments to be clinically transferable, methods of neurotrophin treatment that are safe, biocompatible and can support long-term auditory neuron survival are necessary. Cell transplantation and gene transfer, combined with encapsulation technologies, have the potential to address these issues. This study investigated the survival-promoting effects of encapsulated BDNF over-expressing Schwann cells on auditory neurons in the deaf guinea pig. In comparison to control (empty) capsules, there was significantly greater auditory neuron survival following the cell-based BDNF treatment. Concurrent use of a cochlear implant is expected to result in even greater auditory neuron survival, and provide a clinically relevant method to support auditory neuron survival that may lead to improved speech perception and language outcomes for cochlear implant patients.

## Introduction

Sensorineural hearing loss (SNHL) accounts for 80–90% of all cases of hearing loss, and is a form of deafness associated with loss of the auditory hair cells. Hair cell loss may result from acoustic trauma, ototoxic drugs, cochlear infection, genetic abnormalities or simply through aging. The cochlear implant, or *bionic ear*, is a neural prosthesis which acts to provide auditory cues to patients with a severe-to-profound SNHL by circumventing the damaged or destroyed hair cells to electrically stimulate the primary auditory neurons of the cochlea. However, auditory neurons undergo progressive degeneration in SNHL, and so the rescue of these neurons from deafness-induced degenerative changes may provide clinical benefits in terms of enhancing speech and language outcomes in cochlear implant patients.

Auditory neuron degeneration in deafness occurs, at least partly, because of a loss of endogenous neurotrophic support normally provided by the hair cells [1]–[5] and supporting cells [6] of the organ of Corti. Consequently, treatment of the deafened auditory system with neurotrophic factors, via various delivery methods, is reported to elicit protective effects and prevent, or at least slow, deafness-induced auditory neuron degeneration [7]–[22]. In addition, studies investigating the combined application of neurotrophic factor treatment and chronic electrical stimulation from a cochlear implant electrode array report enhanced auditory neuron survival over either treatment alone [17], [23], as well as functional improvements in terms of reduced electrically-evoked auditory brainstem response thresholds [17], [24]–[26].

However, the current techniques for experimental delivery of neurotrophins into the cochlea remain unsuitable for use in human patients. For example, mini-osmotic pumps have a finite delivery period, and there is also a risk of infection associated with the insertion of a cannula-based system into the cochlea [27]. In addition, the survival effects of neurotrophic factors on auditory neurons are not maintained beyond the treatment period [8], [28], and studies in other neural systems also suggest that the survival effects of neurotrophic factors only last as long as the treatment itself [29], [30]. While it has been reported that auditory neurons can survive for up to two weeks after the cessation of intracochlear BDNF treatment [31], this time-frame is too short to assess if there are any lasting survival effects in the deaf cochlea, as would be required for useful clinical application. Interestingly, ongoing intracochlear electrical stimulation after a short period of neurotrophin treatment prolongs the neurotrophin-induced survival effects beyond the period of neurotrophin treatment [28]. It is therefore important to develop a clinically viable technique for neurotrophin delivery into the cochlea that can be used in conjunction with a cochlear implant, and can lead to long-term or permanent rescue of auditory neurons from the degenerative effects of deafness.

Transplanting neurotrophin-secreting cells into the deaf cochlea is a potential therapeutic option for neurotrophin delivery that addresses the issues around other delivery methods. Numerous cell types, including fibroblasts [32]–[34], Schwann cells [35]–[38] and stem cells [39]–[41] have been successfully genetically modified to express neurotrophins. These cells can elicit positive effects in terms of neuronal survival, regeneration, myelination and recovery of function in various models of neurodegeneration [32]–[38], [40], [41]. Importantly, long-term neurotrophin expression of over 12 months is possible [42].

For clinical application in deafness, cell transplantation is likely to require the use of encapsulation technologies. The semi-permeable membranes of these capsules allow diffusion of oxygen and nutrients into the capsule, and therapeutic agents such as neurotrophic factors and cellular waste out of the capsule [43]–[45]. Encapsulation of cells in a biocompatible matrix would protect the transplanted cells against the immune responses of the host without the use of toxic immunosuppressant drugs, thereby minimising the associated risk of transplant rejection [46], [47]. In addition, in the cochlea, encapsulation would also prevent cellular migration from the site of implantation [48].

A recent study demonstrated greater auditory neuron survival following the implantation of BDNF-expressing fibroblasts, encapsulated in agarose, into the deaf guinea pig cochlea [34]. However, as agarose is a biodegradable substance, it is not known how long such protective effects would last. More stable encapsulation techniques have been developed using non-biodegradable, biocompatible alginate, in which choroid plexus cells were viable for at least six months in the brain of rats [47], and pancreatic islet cells survived for over nine years following intraperitoneal implantation in a human patient [46].

The current study investigated cell-based techniques as a potential clinically applicable means of providing neurotrophic support and promoting auditory neuron survival in deafness *in vivo*. Specifically, we combined cell-based gene transfer with alginate encapsulation technology to assess the survival effects of encapsulated BDNF-expressing Schwann cells on auditory neurons in the deaf guinea pig.

## Methods

### Ethics Statement

All animal experiments were performed in accordance with the Code of Practice For the Care and Use of Animals For Scientific Purposes of the National Health and Medical Research Council of Australia, and the Guide for the Care and Use of Laboratory Animals of the National Institutes of Health, USA. Experiments were performed under the approval of the Animal Research and Ethics Committee of the Royal Victorian Eye and Ear Hospital (Project Number 07/143A), Melbourne, Australia.

### Preparation of BDNF-expressing Schwann cells

#### Schwann cell transfections

Expression plasmids encoding for the reporter gene enhanced green fluorescent protein (EGFP) or C-terminal EGFP-tagged rat prepro BDNF were kindly provided by Dr Volkmar Lessmann, from the Johannes Gutenberg Universität, Mainz, Germany. The BDNF expression vector was constructed by insertion of the complete sequence of rat prepro BDNF cDNA into the cytomegalovirus-promoter driven pEGFP-N1 expression vector (Clontech, Cambridge, UK) as previously described [49]–[51].

Schwann cells were isolated from early postnatal rat sciatic nerve and purified as previously described [52]. The Schwann cells were transfected to over-express either EGFP or EGFP-tagged BDNF using the lipid-based transfection reagent Lipofectamine 2000, as previously described [38]. Briefly, Schwann cells were grown on poly-L-lysine (Sigma-Aldrich, Castle Hill, NSW, Australia) coated 75 cm^2^ flasks in Schwann cell media (SCM; Dulbecco's modified Eagle's medium [DMEM; Thermo Electron Corporation, Noble Park, VIC, Australia] containing 2 mM L-glutamine [Thermo], 50 U/ml penicillin/streptomycin [Thermo], 10% fetal bovine serum [FBS; Thermo], 0.08% bovine pituitary extract [Sigma-Aldrich] and 2 µM Forskolin [Sigma-Aldrich]), at 37°C, 10% CO_2_. On the day prior to transfection, Schwann cells were sub-cultured into poly-L-lysine coated 6-well plates at a concentration of 2×10^5^ cells/well, ensuring cells would be in the log phase of differentiation on the day of transfection.

The Lipofectamine 2000 (Invitrogen, Melbourne, VIC, Australia) reagent was prepared as per manufacturer's guidelines, with 4 µg DNA used for each well of Schwann cells. Schwann cells were rinsed with phosphate buffered saline (PBS), and fresh SCM was added to each well. The DNA/lipid complex was added to each well of Schwann cells and mixed by gently rocking the plate. The plates were then incubated at 37°C, 10% CO_2_ overnight.

The following day, the presence of the EGFP reporter gene under direct fluorescence microscopy was used to confirm successful transfection. Schwann cells were sub-cultured and after a further 24 hours selection of stable transformants commenced with the addition of geneticin (G418 sulphate, 400 µg/ml; Invitrogen). Following two weeks of selective pressure the cells were purified by fluorescence activated cell sorting and the resultant BDNF-Schwann cells and control EGFP-Schwann cells were maintained under selective conditions (200 µg/ml geneticin) and sub-cultured every 3–4 days.

Conditioned media was collected at weekly intervals for up to four weeks, and the concentration of BDNF secreted by the BDNF-Schwann cells was determined via enzyme linked immunosorbant assay (ELISA) analysis of conditioned media using an Emax Immunoassay System kit (Promega, Annandale, NSW, Australia) (n≥8).

#### Encapsulation

**Figure 1:**
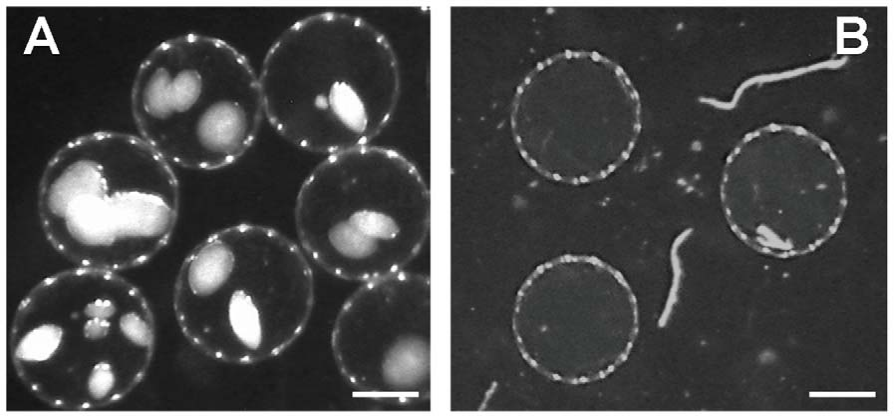
Alginate capsules *in vitro*. (A) Alginate capsules containing BDNF-Schwann cells and (B) empty control capsules. Capsules are 500–600 µm in diameter. Scale bar = 250 µm.

The BDNF-Schwann cells were encapsulated in a biocompatible matrix (Immupel™) courtesy of our collaborative partner, Living Cell Technologies Limited [47]. Briefly, a single cell suspension of BDNF-Schwann cells was mixed with a 1.7% solution of alginate at a ratio of 1.72×10^6^ cells/mL alginate, and this mixture was pumped through a fine aperture nozzle into a bath of calcium chloride (1.2%). The resulting gelled beads were washed and serially coated with poly-L-ornithine (0.1%), poly-L-ornithine (0.05%) and alginate (0.17%), and were then washed in saline and treated with sodium citrate to chelate calcium and thus liquefy the intra-capsular alginate. The resultant capsules were 500–600 µm in diameter, and contained approximately one thousand BDNF-Schwann cells (Figure 1A). Empty alginate capsules were prepared in the same way, but without the cells (Figure 1B).

### *In vivo* procedures

#### Ototoxin-induced deafening

A total of 23 pigmented guinea pigs of both sexes (400–600 g) were used for this study. Animals were anaesthetised with a combination of intramuscular (i.m.) ketamine (40 mg/kg; Parnell Laboratories, Alexandria, NSW, Australia) and xylazine (4 mg/kg; Troy Laboratories, Smithfield, NSW, Australia). All animals had otoscopically normal tympanic membranes and normal hearing was confirmed by click-evoked auditory brainstem responses (ABRs) with thresholds of <43 decibels peak equivalent sound pressure level (dB p.e. SPL) [17]. One week later, animals were anaesthetised using 3–4% isoflurane gas (APS Specialty Chemicals, Seven Hills, NSW, Australia) delivered in 100% oxygen at 1 L/kg/min for induction and 1–1.5% isoflurane in 1 L/kg/min oxygen for maintenance. Guinea pigs were deafened via an intravenous infusion of the loop diuretic frusemide (130 mg/kg; Troy Laboratories) followed by a subcutaneous (s.c.) injection of the ototoxic aminoglycoside kanamycin sulphate (520 mg/kg; Sigma-Aldrich).

#### Implantation surgery

Five days post-deafening, the animals were anaesthetised with ketamine (60 mg/kg, i.m.) and xylazine (4 mg/kg, i.m.) and ABRs were performed to confirm ototoxin-induced deafness. All animals exhibited a severe-profound SNHL, as indicated by click-evoked ABR thresholds of >93 dB p.e. SPL in both ears. Animals then underwent surgery using our standard surgical techniques [8], [28]. Briefly, under aseptic conditions, a postauricular incision was made behind the left ear to expose the tympanic bulla. A 1 mm cutting burr was used to open the bulla and enable visualisation of the basal turn of the cochlea, and a 0.6 mm diamond drill piece was used to make a cochleostomy, approximately 1 mm in diameter, in the scala tympani at the level of the basal turn. Alginate capsules (10–12) containing BDNF-Schwann cells (eBDNF-SC cohorts) or empty capsules (control cohorts) were slowly injected into the cochlea via a cannula. The cochleostomy was sealed with muscle and the wound closed in two layers. The animals survived for experimental periods of two weeks (2 w) or four weeks (4 w) post-implantation. The experimental cohorts are summarised in Table 1.

**Table 1:** Experimental Cohorts.

Cohort	Treatment	Experimental period	Number of animals
eBDNF-SC-2 w	Encapsulated BDNF-Schwann cells	2 weeks	5
eBDNF-SC-4 w	Encapsulated BDNF-Schwann cells	4 weeks	6
Control-2 w	Empty capsules	2 weeks	6
Control-4 w	Empty capsules	4 weeks	6

Summary of experimental cohorts used in the study. Animals received implantations of either encapsulated BDNF-Schwann cells (eBDNF-SC) or empty capsules (control), and survived for either two or four weeks post-implantation.

#### Histology

Following the experimental period, the animals were euthanized with an intraperitoneal injection of pentobarbitone sodium (160 mg/kg; Troy Laboratories) and intracardially perfused with heparinised normal saline followed by 10% neutral buffered formalin. The cochleae were harvested and decalcified in 10% ethylenediamine-triacetic acid in 0.1 M phosphate buffer. The cochleae were then embedded in OCT Compound (Tissue-Tek; ProSciTech, Thuringowa, Qld, Australia) and sectioned on a cryostat at 12 µm. Sections were stained with haematoxylin and eosin.

### Analysis and Statistics

BDNF expression by BDNF-Schwann cells and control (EGFP) Schwann cells was quantified by ELISA analysis of conditioned media and expressed as picograms of BDNF per day, per million cells. Statistical differences in the amount of BDNF produced over time were identified using a one-way analysis of variance (ANOVA), and Dunn's Method was used for multiple comparisons. A difference was considered statistically significant at *P*<0.05.

Auditory neuron survival was quantified in three representative, non-consecutive mid-modiolar sections for each experimental animal using a Zeiss microscope and Axiovision software. The absolute number of surviving auditory neurons with a clear nucleus and nucleolus were counted and the cross-sectional area of Rosenthal's canal from lower basal to the cochlear apex was measured. Auditory neuron density (neurons/mm^2^) was calculated for each cochlear region from lower basal to the apex, and the data is presented as an average across all of these regions. Results are expressed as mean ± standard error of the mean (SEM). Due to the surgical procedures involved, any protection afforded by the eBDNF-SCs is best controlled for by the empty control cohort. Therefore, a two-way ANOVA was used to determine the effects of both treatment (eBDNF-SC versus control) and treatment duration (2 w versus 4 w), with data considered statistically significant if *P*<0.05.

## Results

### Over-expression of BDNF by Schwann cells

**Figure 2:**
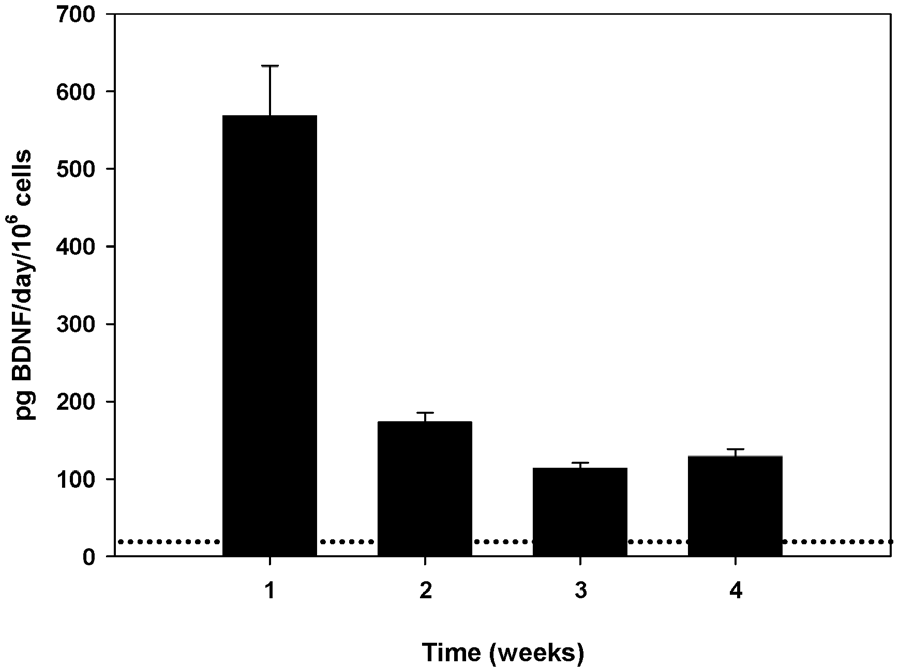
BDNF expression by BDNF-Schwann cells post-transfection. The amount of BDNF produced by the BDNF-Schwann cells was quantified by ELISA analysis of conditioned media. BDNF expression was greatest one week after transfection, but after four weeks was still significantly greater (*P*<0.05) than that of control Schwann cells expressing EGFP only (indicated by dotted line).

Fluorescence microscopy confirmed that Schwann cells were successfully transfected to over-express BDNF, with transfected cells appearing green due to the presence of the EGFP reporter gene. In addition, successful transfection was confirmed by ELISA analysis of conditioned media. Immediately post-transfection, the BDNF-Schwann cells produced a significantly greater amount of BDNF (568.80±64.34 pg/day/10^6^ cells [mean ± SEM]) than control EGFP-Schwann cells (12.12±3.73 pg/day/10^6^ cells) (*P*<0.001). There was a large and significant (*P*<0.001) decrease in BDNF production in the second week post-transfection (172.89±12.85 pg/day/10^6^ cells), although this was still significantly more BDNF than that produced by the control EGFP-Schwann cells, and BDNF expression by the BDNF-Schwann cells remained significantly greater than controls for the four week post-transfection period (*P*<0.05; Figure 2).

### Implantation of alginate capsules

**Figure 3:**
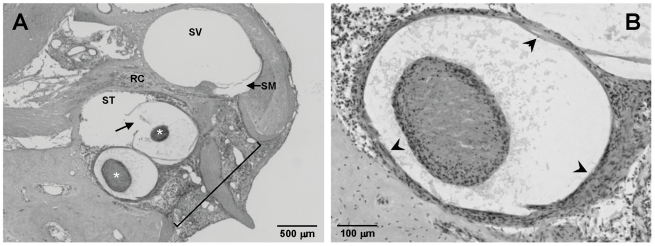
Alginate capsules *in vivo*. (A) Histological section showing two alginate capsules containing clusters of BDNF-Schwann cells (asterix), in the basal region of the deaf guinea pig cochlea. One capsule was damaged during the histological processing, leading to rupture of the capsule wall (arrow). The large cochleostomy required for insertion of the capsules is outlined, and a vigorous tissue response is apparent in this area. It should be noted that the tissue response was typically localised to the region of the cochleostomy and there was no fibrous tissue in the more apical regions of the cochlea. (B) A higher magnification image showing a capsule containing BDNF-Schwann cells within the basal turn of the deaf guinea pig cochlea. The capsule walls are indicated by arrowheads. ST = scala tympani; SM = scala media; SV = scala vestibuli; RC = Rosenthal's canal.

Ototoxically deafened guinea pigs were divided into four groups and implanted with alginate capsules containing BDNF-Schwann cells (eBDNF-SC) or empty capsules (control) for either two weeks (2 w) or four weeks (4 w). Figure 3 shows histological images of encapsulated BDNF-Schwann cells implanted into the basal turn of the cochlea. The capsules were 500–600 µm in diameter and contained clusters of BDNF-Schwann cells. Histological processing led to some distortion of the spherical shape of the capsules, as well as some damage to the capsule wall (Figure 3A, arrow). We also observed some tissue response in both eBDNF-SC and control capsule cohorts, which was typically localised to the region of the cochleostomy. Importantly, there was no fibrous tissue in the more apical regions of the cochlea. A cluster of encapsulated BDNF-Schwann cells *in situ* can be seen at higher magnification in Figure 3B.

### Survival effects of encapsulated BDNF-Schwann cells on auditory neurons in vivo

**Figure 4:**
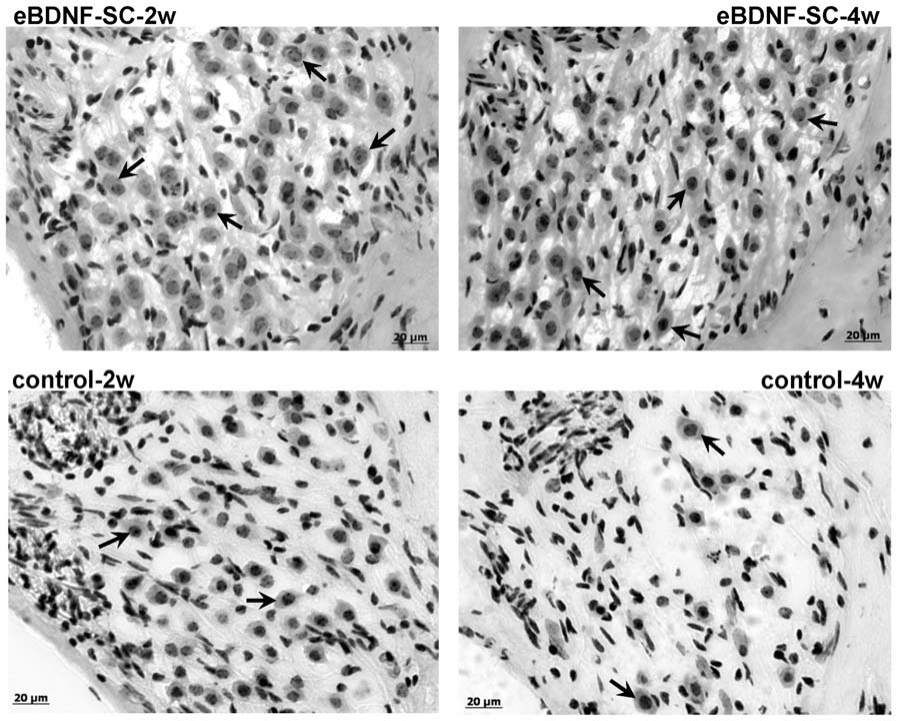
Photomicrographs showing auditory neurons in Rosenthal's canal for each of the experimental cohorts. Histological sections show Rosenthal's canal in the lower basal turn of the deaf guinea pig cochlea implanted with either encapsulated BDNF-Schwann cells or empty control capsules, for either two or four weeks. Auditory neurons were identified and counted based upon the presence of an identifiable cell soma and a clear nucleus and nucleolus. Examples are indicated by arrow heads. There are more auditory neurons in cochleae implanted with encapsulated BDNF-Schwann cells than in cochleae receiving empty capsule control implants.

Ototoxin exposure led to a complete loss of the sensory epithelium of the organ of Corti, which was apparent in all animals of each treatment group. Histological images showing auditory neuron survival in Rosenthal's canal of the lower basal turn of the deaf guinea pig cochlea, for each of the experimental cohorts, are shown in Figure 4. Enhanced auditory neuron survival is apparent in both eBDNF-SC cohorts (2 w and 4 w) compared to the time-matched controls. In addition, the auditory neurons in the eBDNF-SC treated cochleae displayed morphological and histological characteristics typical of healthy cells, with round cell bodies and identifiable nuclei and nucleoli.

**Figure 5:**
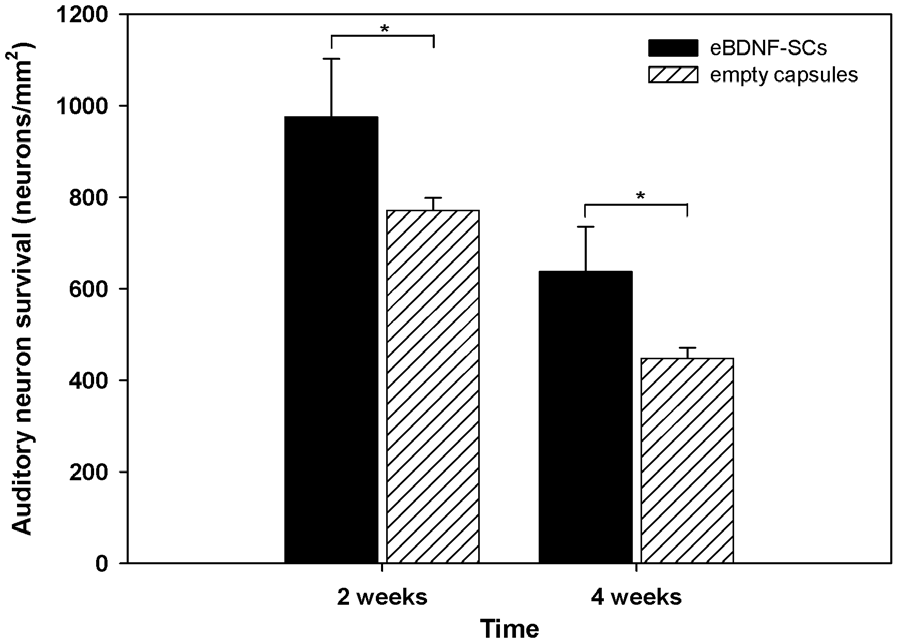
The effects of cell-based neurotrophin treatment on auditory neuron survival in the deaf guinea pig. Implantation of encapsulated BDNF-Schwann cells (eBDNF-SCs) resulted in enhanced auditory neuron survival in comparison to empty capsules, over both two and four weeks (* *P*<0.05). The number of surviving auditory neurons was calculated as an average across all cochlear turns.

Figure 5 illustrates auditory neuron density, averaged across the cochlea from base to apex, for all experimental cohorts. Auditory neuron survival in the control-2 w cohort was 770.97±27.20 neurons/mm^2^ (mean ± SEM). In comparison, auditory neuron survival in the eBDNF-SC-2 w cohort was significantly greater (*P*<0.05) at 975.37±127.02 auditory neurons/mm^2^, which is a 26% increase in survival following the implantation of encapsulated BDNF-Schwann cells. A significant (*P*<0.05) rescue effect was also observed in the eBDNF-SC-4 w cohort (637.02±98.55 neurons/mm^2^) in comparison to the control-4 w group (447.8±23.27 neurons/mm^2^), which was equivalent to a 42% increase in auditory neuron survival in the eBDNF-SC treated cochleae. A two-way ANOVA found there was a main effect of ‘treatment’ (*P*<0.05), indicating that cell-based BDNF treatment enhanced auditory neuron survival in comparison to empty control capsules. There was also a main effect of ‘treatment duration’ (*P*<0.001) with a greater proportion of auditory neuron rescue in the four-week cohort (42%) versus the two-week cohort (26%), despite the continued auditory neuron degeneration with the longer period of deafness. There was no interaction between treatment and treatment duration (*P* = 0.343).

## Discussion

The current study is the first to use *ex vivo* neurotrophin treatment in conjunction with non-biodegradable cell encapsulation to deliver BDNF into the cochlea and promote auditory neuron survival in the deaf guinea pig.

These findings demonstrate that cell-based neurotrophin treatment is a viable option for supporting auditory neuron survival in deafness. Implantation of eBDNF-SCs resulted in 975.37±127.02 surviving auditory neurons/mm^2^ after two weeks, and 637.02±98.55 neurons/mm^2^ after four weeks. Previously, we have reported auditory neuron density in the normal hearing guinea pig to range between approximately 800–950 neurons/mm^2^ [17], [22]. This indicates that auditory survival after two weeks of cell-based BDNF treatment is similar to that seen in normal hearing animals. These findings are therefore an important step in the path to developing a clinically transferable technique for the delivery of neurotrophins to the deaf cochlea.

It is hypothesised that preventing auditory neuron degeneration in deafness will improve the benefits to, and outcomes for, cochlear implant patients. However, the majority of neurotrophin delivery techniques used in previous studies are not suitable for use in human patients [27], [53]. For example, intracochlear cannula-based infusion has a significant risk of infection that is unacceptable for human cochlear implant recipients. Furthermore, osmotic pump-based systems have a finite drug reservoir and neurotrophin-induced auditory neuron survival is not maintained beyond the treatment period [8]. In addition, the use of alternative drug delivery systems [54], [55] may be precluded by the long-term bioactivity of neurotrophins under physiological conditions, which is currently unknown. The application of neurotrophic factors via application on and diffusion through the round window membrane, while appealing due to the non-invasive and atraumatic nature of the procedure, provides only short-term delivery [56] and may be compromised by non-uniform distribution through the cochlea [57], [58] as well as variability in membrane permeability due to cochlear pathologies [59].

Therefore, cell-based delivery of neurotrophins has the potential to overcome these issues by eliminating the extracochlear elements that could facilitate the transmission of infection into the cochlea, while providing continuous production and secretion of neurotrophic factors. In addition, cells genetically modified to express neurotrophins can do so long-term, for ≥13 months [42], and so these techniques have the potential to achieve long-term neurotrophin secretion and long-term or possibly permanent rescue of auditory neurons from deafness-induced degeneration.

Cell-based neurotrophin treatment meets a number of other important criteria for clinical application in the cochlea. Firstly, neurotrophin delivery is more physiologically relevant in terms of amount and dose rate. The potential side effects of excessive neurotrophin treatment, as may occur with pump-based systems, is currently unknown, but may be significant since, in addition to neuronal survival and differentiation effects, neurotrophins can also elicit changes in ion channel properties and neurotransmitter release, and are important in modulating activity-dependent neuronal plasticity that is essential for the functional and structural refinement of neuronal circuits [60], [61].

Importantly, cell-based BDNF treatment in this study resulted in similar auditory neuron survival after four weeks to that described previously using mini-osmotic pump infusion of BDNF [9], despite the large difference in the amount of neurotrophin delivered. Therefore, effective auditory neuron survival can be achieved using cell-based neurotrophin treatment, further supporting such delivery methods as a viable means of providing neurotrophic support to promote auditory neuron survival in deafness.

A further consideration for the clinical application of cell-based therapies in the cochlea is the potential migration or dispersal of cells from the site of implantation, given the patency of the cochlear aqueduct with the cerebrospinal fluid of the central nervous system. The implantation of cells in suspension into the fluid-filled spaces of the cochlea can result in migration of the cells throughout the cochlea and into the vestibular organs [48], and unilateral viral inoculation of the cochlea also led to gene expression in the contralateral cochlea and CNS [62], [63]. The predisposition of migration or spread from the cochlea due to the presence of the cochlear aqueduct is therefore an important safety consideration for implanting cells into the cochlea, which can be prevented by the use of cell encapsulation.

A further feature of encapsulation technologies is protection against immunological responses without the use of toxic immunosuppressant drugs. This is especially important in xenotransplantation studies, such as the current study where rat cells were transplanted into the guinea pig. The alginate encapsulation technique used in this study is reported to be biocompatible and well tolerated following implantation into various species, including human, and for up to nine years [46]. These studies also demonstrate that this encapsulation technique is suitable for long-term survival of the encapsulated cells. While we did see some tissue response in the current study following surgery (Figure 3), this was observed in both eBDNF-SC and control capsule cohorts, and was localised to the region of the cochleostomy. Previously, in studies using cannula insertion and mini-osmotic pump delivery of neurotrophins, we did not observe a fibrous tissue reactions [8], [9]. The tissue response we observed in the present study is most likely due to the large cochleostomy that was required for the insertion of the capsules, rather than a reaction to the capsules themselves. Despite the small size of the capsules (500–600 µm diameter), they were much larger than the cannula used in mini-osmotic pump studies (∼160 µm diameter), and as a result the size of the cochleostomy required for capsule implantation was significantly greater, and may have elicited a tissue response due to structural damage to the wall of the cochlea. Furthermore, a muscle plug was used to seal the cochleostomy, which may have also initiated a fibrous reaction within the fluid-filled spaces of the cochlea. Regardless of the cause, fibrous tissue reactions, however mild, are undesirable within the cochlea for a number of reasons [17]. In cochlear implant patients fibrous tissue will lead to increased electrode impedance and consequently increased power consumption, thereby reducing the efficacy of the device. Neurotrophin diffusion and efficacy may also be impacted by fibrous tissue reactions. Future studies using encapsulated cell transplantation may utilise a round window approach for implantation to minimise such effects.

The size of the capsules may also limit depth of insertion into the cochlea. The scala tympani of the guinea pig cochlea has a cross-sectional area of approximately 1300 µm^2^ at its widest point near the base, and narrows to approximately 500 µm^2^ within 4 mm from the round window [64]. These dimensions indicate the insertion of capsules into the guinea pig cochlea will be restricted to the basal region, consistent with what we observed in this study. The human scala tympani is much larger than that of the guinea pig, almost 3000 µm^2^ at its widest point basally [65], although insertion of capsules in the human cochlea would still be restricted to the base and middle turns.

Despite the localisation of the capsules to the basal cochlear region near the site of implantation, we did not observe any localised survival effects (data not shown). This is also consistent with some of our previous work [8], [9], and suggests that the BDNF was effectively distributed throughout the cochlea. However, a recent study implanting BDNF-expressing fibroblasts, encapsulated in agarose, into the cochlea of deaf guinea pigs demonstrated that auditory neuron survival was greater in the vicinity of the basal turn, adjacent to the implant site [34]. Similarly, it has been reported that auditory neuron survival following neurotrophin treatment and chronic electrical stimulation was greatest in the basal region, adjacent to the implantation site [17]. These differences may be a result of the different experimental techniques used, as compared to the current study. For example, in the study by Rejali et al. (2007) in which the BDNF-expressing fibroblasts were seeded onto an electrode array, the effects may have been limited to the insertion depth into the cochlea, and the agarose may have reduced the diffusion of BDNF through the cochlea [34]. Similarly, the increased auditory neuron survival seen basally following BDNF treatment and chronic electrical stimulation may reflect the localised current distribution from the electrode in that model [17].

The present study has utilised encapsulation technologies to provide cell-based BDNF treatment to the deaf cochlea to support auditory neuron survival. While this provides proof-of-concept that such a technique is clinically applicable, further studies are required that address the issue of long-term auditory neuron survival in deafness. This will require the use of cells that are confirmed to secrete the desired neurotrophins for extended periods of time, and that are implanted for longer than four weeks, preferably in conjunction with a cochlear implant.

### Conclusion

The findings from this study suggest that cell-based neurotrophin treatment, incorporating encapsulation technologies, provides a clinically transferable therapeutic option for the delivery of neurotrophic factors to reduce or prevent auditory neuron degeneration in sensorineural hearing loss. Long-term studies investigating the combined application of cell-based neurotrophin treatment and chronic electrical stimulation from a cochlear implant are expected to further enhance auditory neuron survival in the deaf cochlea, thereby enhancing and extending the benefits of the cochlear implant.
